# Supplementary material for: Astrocytic Nrf2 Mediates the Neuroprotective and Anti-Inflammatory Effects of Nootkatone in an MPTP-Induced Parkinson’s Disease Mouse Model
Source: Antioxidants (Basel). 2023 Nov 13;12(11):1999. doi: 10.3390/antiox12111999 (PMC10669233; doi:10.3390/antiox12111999)
Supplement: Supplementary file 1 [file antioxidants-12-01999-s001.zip › Supplementary Figures.pdf]

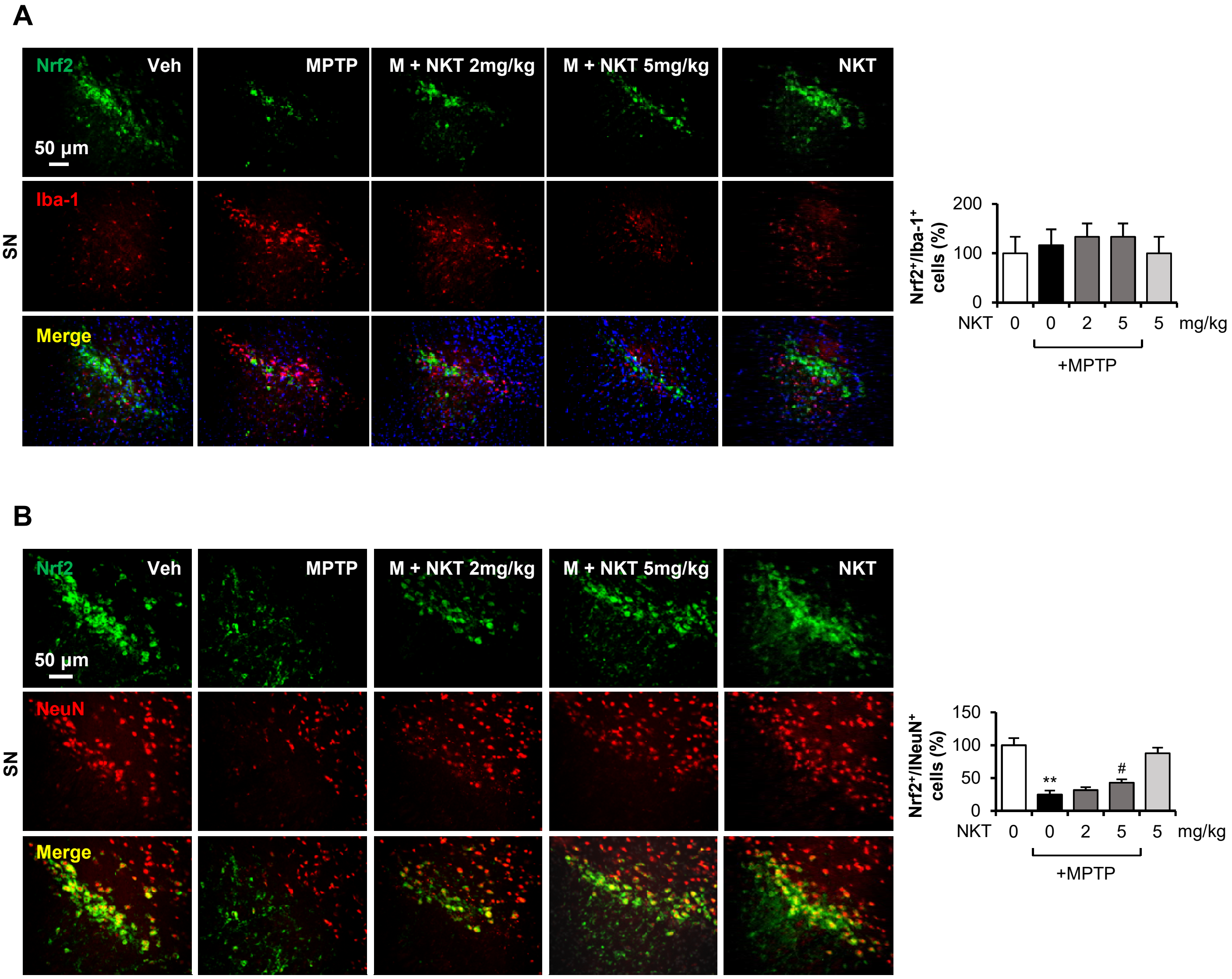

Figure S1. Effects of NKT on microglial/neuronal Nrf2 expression in the brains of MPTP-treated mice. (A) IF staining data showing Nrf2 and Iba-1 expression in the SN of MPTP mice (n = 6–7, three sections per brain). Representative images are provided in the left panel, and quantification of Nrf2^{+}/Iba-1^{+} cells is shown in the right panel. (B) IF staining showing Nrf2 and NeuN expression in the SN of MPTP mice (n = 6–7, three sections per brain). Representative images are shown in the left panel, and quantification of Nrf2^{+}/NeuN^{+} cells is shown in the right panel. Data are presented as the mean ± SEM. $^{**}p < 0.01$ vs. control group; $^{\#}p < 0.05$ vs. MPTP-treated group.

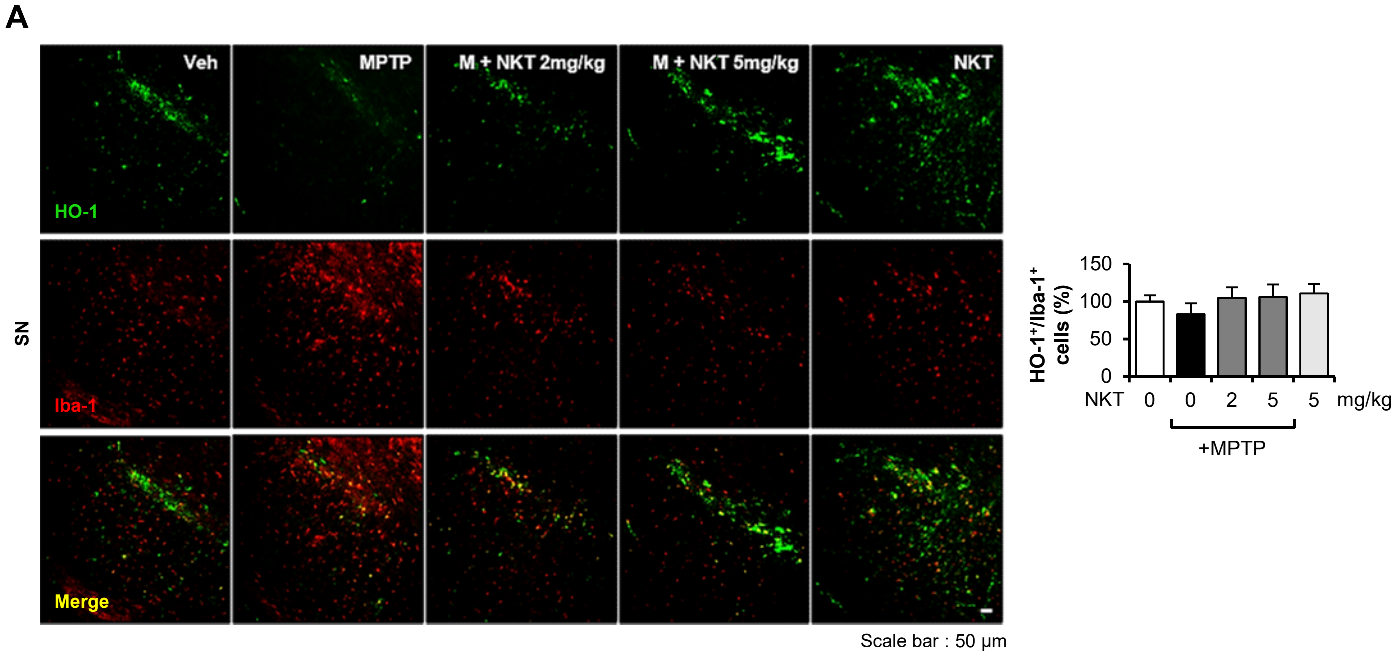

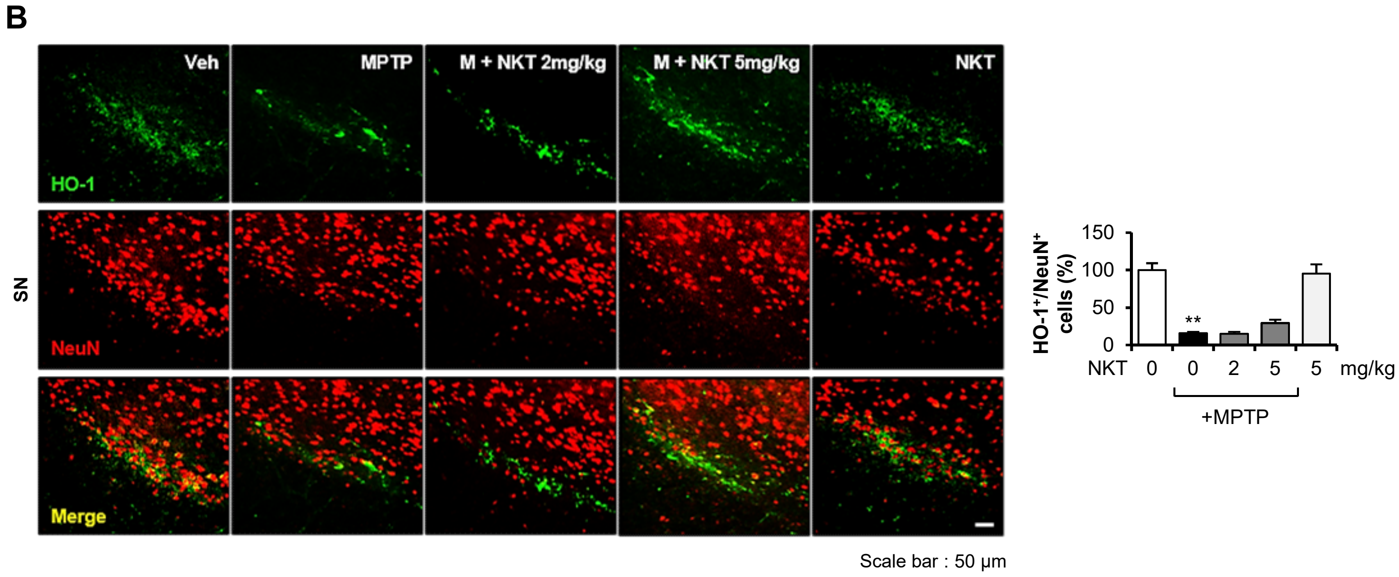

Figure S2. Effects of NKT on microglial/neuronal HO-1 expression in the brains of MPTP-treated mice. (A) IF staining data showing HO-1 and Iba-1 expression in the SN of MPTP mice (n = 6–7, three sections per brain). Representative images are provided in the left panel, and quantification of HO-1$^+$/Iba-1$^+$ cells is shown in the right panel. (B) IF staining results showing HO-1 and NeuN expression in the SN of MPTP mice (n = 6–7, three sections per brain). Representative images are provided in the left panel, and quantification of HO-1$^+$/NeuN$^+$ cells is shown in the right panel. Data are presented as the mean ± SEM. $^{**}p < 0.01$ vs. control group.

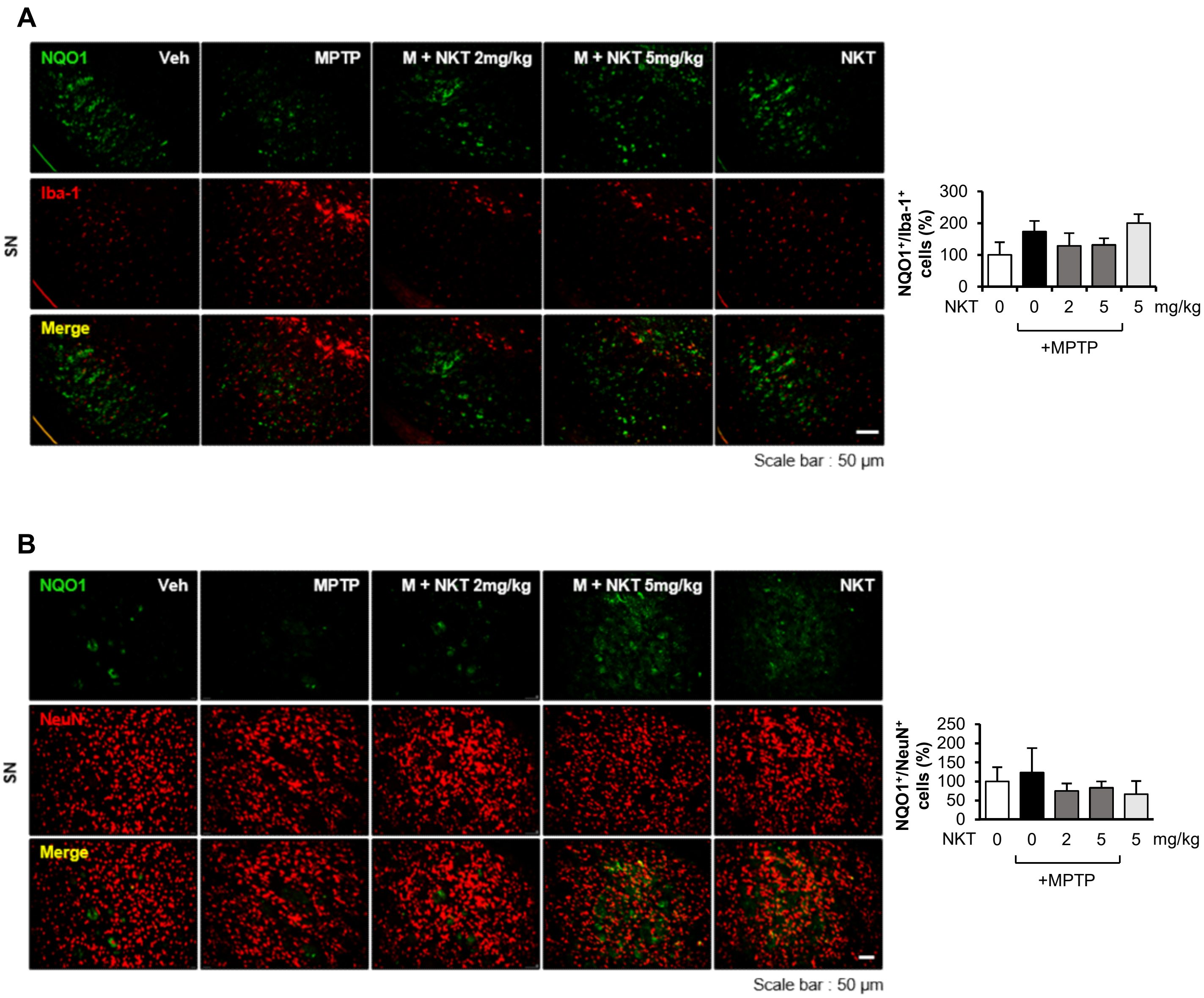

Figure S3. Effects of NKT on microglial/neuronal NQO1 expression in the brains of MPTP-treated mice. (A) IF staining results showing NQO1 and Iba-1 expression in the SN (n = 6–7, three sections/brain). Representative images are shown in the left panel, and quantification of NQO1$^+$/Iba-1$^+$ cells is shown in the right panel. (B) IF staining results showing NQO1 and NeuN expression in the SN of MPTP mice (n = 6–7, three sections per brain). Representative images are shown in the left panel, and quantification of NQO1$^+$/NeuN$^+$ cells is shown in the right panel. Data are presented as the mean ± SEM.
