# Supplementary material for: Astrocytic Nrf2 Mediates the Neuroprotective and Anti-Inflammatory Effects of Nootkatone in an MPTP-Induced Parkinson’s Disease Mouse Model
Source: Antioxidants (Basel). 2023 Nov 13;12(11):1999. doi: 10.3390/antiox12111999 (PMC10669233; doi:10.3390/antiox12111999)
Supplement: Supplementary file 1 [file antioxidants-12-01999-s001.zip › Supplementary Tables.pdf]

**Table S1.** List of primary antibodies used in western blot analysis

| Antigen | Manufacturer | Catalog number | Dilution |
|---|---|---|---|
| TH | Cell Signaling | 58844 | 1:2000 |
| p-CREB | Cell Signaling | 9198 | 1:2000 |
| NQO1 | Cell Signaling | 62262 | 1:5000 |
| Bcl2 | Abcam | ab59348 | 1:5000 |
| GDNF | Abcam | ab18956 | 1:2000 |
| Iba-1 | Wako | 019-19741 | 1:2000 |
| iNOS | BD bioscience | 610431 | 1:2000 |
| TNF-α | Santa Cruz | 133192 | 1:500 |
| IL-6 | Santa Cruz | 57315 | 1:500 |
| TLR2 | Santa Cruz | 10739 | 1:2000 |
| TLR4 | Santa Cruz | 10741 | 1:2000 |
| IL-10 | Santa Cruz | 365858 | 1:500 |
| Nrf2 | Santa Cruz | 722, 365949 | 1:500 |
| TGF-β | Santa Cruz | 146 | 1:500 |
| PGC-1α | Millipore | ST1202 | 1:1000 |
| HO-1 | Enzo Life | ADI-SPA-895 | 1:5000 |
| MnSOD | Enzo Life | ADI-SOD-111 | 1:5000 |
| 4-HNE | Alpha diagnostic | HNE11-S | 1:5000 |
| COX-2 | MyBioSource | MBS9410607 | 1:2000 |
| IL-1β | MyBioSource | MBS175705 | 1:2000 |
| GCLC | MyBioSource | MBS9132879 | 1:5000 |
| GCLM | GeneTex | GTX114075 | 1:5000 |
| GFAP | Sigma-Aldrich | G3893 | 1:5000 |
| BDNF | Sigma-Aldrich | SAB2108004 | 1:2000 |
| β-actin | Sigma-Aldrich | A1978 | 1:10000 |

**Table S2.** List of primary antibodies used in IHC and IF staining

| Antigen | Manufacturer | Catalog number | Dilution |
|---|---|---|---|
| TH | Cell Signaling | 58844 | 1:2000 |
| GFAP | Sigma-Aldrich | G3893 | 1:5000 |
| Iba-1 | Wako | 019-19741 | 1:2000 |
| NeuN | Millipore | MAB377 | 1:2000 |
| Nrf2 | Santa Cruz | 722 | 1:1000 |
| HO-1 | Enzo Life | ADI-SPA-895 | 1:5000 |
| NQO1 | Cell Signaling | 62262 | 1:5000 |
| 8-OHdG | Antibodies-online | ABIN674319 | 1:1000 |
